# Identification of bacterial communities in sediments of Poyang Lake, the largest freshwater lake in China

**DOI:** 10.1186/s40064-016-2026-7

**Published:** 2016-04-01

**Authors:** Wenbo Kou, Jie Zhang, Xinxin Lu, Yantian Ma, Xiaozhen Mou, Lan Wu

**Affiliations:** College of Life Science, Nanchang University, No. 999, Xuefu da Road, Hongutang New District, Nanchang, 300031 Jiangxi China; Collaborative Innovation Center for Poyang Lake Basin Green Development and Water Security, Nanchang University, Nanchang, 330031 China; Department of Biological Sciences, Kent State University, No. 800 E. Summit Street, Kent, OH 44240 USA

**Keywords:** Bacterial community, Sediment, Poyang Lake, High-throughput sequencing

## Abstract

Bacteria play a vital role in various biogeochemical processes in lacustrine sediment ecosystems. This study is among the first to investigate the spatial distribution patterns of bacterial community composition in the sediments of Poyang Lake, the largest freshwater lake of China. Sediment samples were collected from the main basins and mouths of major rivers that discharge into the Poyang Lake in May 2011. Quantitative PCR assay and pyrosequencing analysis of 16S rRNA genes showed that the bacteria community abundance and compositions of Poyang Lake sediment varied largely among sampling sites. A total of 25 phyla and 68 bacterial orders were distinguished. *Burkholderiales*, *Gallionellales* (*Beta*-*proteobacteria*), *Myxococcales*, *Desulfuromonadales* (*Delta*-*proteobacteria*), *Sphingobacteriales* (*Bacteroidetes*)*, Nitrospirales* (*Nitrospirae*), *Xanthomonadales* (*Gamma*-*proteobacteria*) were identified as the major taxa and collectively accounted for over half of annotated sequences. Moreover, correlation analyses suggested that higher loads of total phosphorus and heavy metals (copper, zinc and cadmium) could enhance bacterial abundance in the sediment.

**Electronic supplementary material:**

The online version of this article (doi:10.1186/s40064-016-2026-7) contains supplementary material, which is available to authorized users.

## Background

Freshwater lakes are one of the most extensively altered ecosystems on earth due to changes of climate, hydrologic flow and human activities related processes, such as land-use and nutrient inputs (Carpenter et al. 2011). Lake sediments are important grounds for series of biogeochemical transformations of essential nutrients (carbon, nitrogen and phosphorus) and contaminants (Nealson 1997; Bouskill et al. 2010). Sediment microorganisms, especially bacteria, play a dominant role in these critical processes. Bacteria-mediated transformations in sediments lead to active exchange of energy and materials with the water column and intimately connect sedimentary processes with diverse aquatic ecosystem functions (Ranjard et al. 2000; Urakawa et al. 2000).

Bacterial community composition (BCC) in freshwater lakes has been extensively investigated, partly because its potentials in predicting major biogeochemical functions. Early studies have shown that lake sediment BCC may be shaped by physicochemical factors, such as temperature, stream flow (Bernhard et al. 2005), pH (Lindström et al. 2005) and nutrient concentrations (Bai et al. 2012; Zhang et al. 2015). In addition, BCC has been found to co-vary with metal concentrations in lake sediments (Cummings et al. 2003; Bouskill et al. 2010; Sauvain et al. 2014). However, the above studies and most available reports were obtained based on investigations of multiple isolated lakes. The relationship between BCC and environmental conditions within individual freshwater lakes, especially those with large volumes and surface areas has not been fully understood (Yannarell and Triplett 2004; Bouzat et al. 2013). Alternatively, whether environmental factors apply similar impacts on BCC in main lake area and estuarine zone remain unclear.

**Fig. 1:**
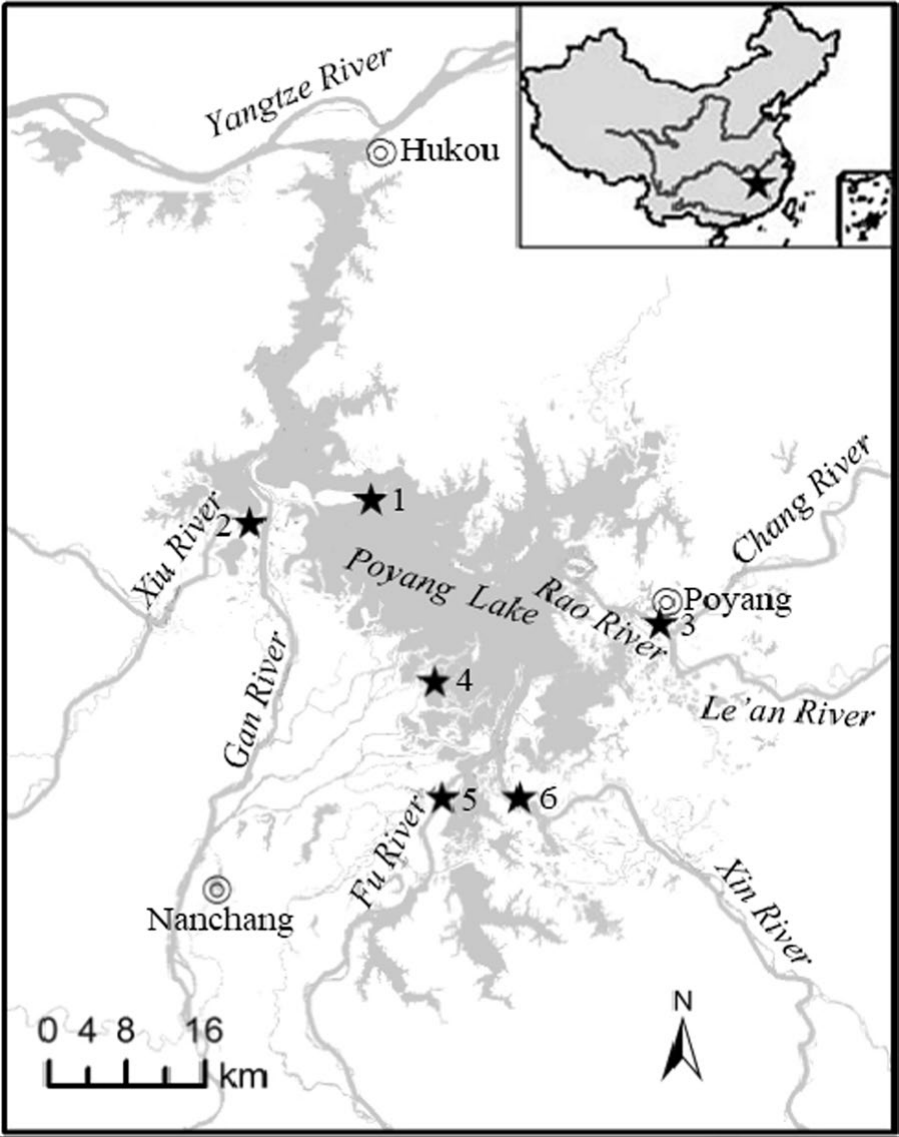
Sampling sites in Poyang Lake. *1* Songmenshan Region, *2* Xiu River Estuary, *3* Rao River Estuary, *4* Nanjishan Region, *5* Fu River Estuary, *6* Xin River Estuary

Poyang Lake (28°52′21″–29°06′46″N, 116°10′24″–116°23′50″E), located in northern Jiangxi Province, is the largest freshwater lake in China with a storage capacity of 2.95 billion m^3^ (Fig. 1). The lake covers an area of 4125 km^2^ and has an average depth of 5.1 m. Poyang Lake is a throughput type of lake, mainly collect freshwater from tributary rivers, including the Gan, Fu, Xiu, Xin and Rao Rivers, and discharging into the Yangtze River. In recent years, natural and anthropogenic inputs of nutrients and xenobiotics have consistently increased. As a result, a decreasing gradient of nutrients and heavy metals along transects from estuaries to main lake basins has established (Liu et al. 2012; Zhang et al. 2014; Wang and Liang 2015). This may result in spatial variations of sediment BCCs and their biogeochemical activities, which may in turn impact the lake ecosystem function. However, so far no study has been done on sediment BCC in Poyang Lake.

In this study, 16S rRNA gene-based quantitative PCR and pyrosequencing were used to examine sediment BCCs in Poyang Lake. Our specific goals were to (1) investigate horizontal dynamics of BCCs (i.e., relative abundance and diversity), (2) examine the potential correlations between BCCs and environmental factors.

## Methods

### Study sites and sample collection

Surface sediments (0–5 cm) were collected in May 2011 from six sites in Poyang Lake (Fig. 1). These sites were chosen to cover both of the main basins of the lake and the mouths of major rivers that discharge into Poyang Lake, including two sites from main basin of the lake, i.e., Site 1 in Songmenshan Region (29°12′26.9″N, 116°11′28.0″E), and Site 4 in Nanjishan Region (28°55′00.1″N, 116°16′44.4″E); four sites from the lake estuaries (mouths of influx rivers), i.e., Site 2 (29°11′27.4″N, 116°00′33.3″E), Site 3 (28°59′10.6″N, 116°40′10.6″E), Site 5 (28°39′54.4″N, 116°9′31.9″E) and Site 6 (28°43′42.2″N, 116°24′34.8″E) from Xiu, Rao, Fu and Xin River to Poyang Lake, respectively.

At each sampling site, triplicate surface sediment samples were taken with a grab sampler to obtain a total of 18 samples. Samples were transferred into sterile polyethylene ziplock bags, put on ice and immediately transported to laboratory. Large organic debris was removed from the sediments with sterile forceps. Afterwards, samples were divided into two aliquots. One aliquot was processed immediately for measurements of sediment property variables; and the other aliquot was stored in sterile polypropylene tubes at −80 °C for molecular analysis.

### Measurement of sediment properties

Sediment pH was measured on sediment slurry at a 1:2.5 (w/v) sediment: distilled water ratio using a FE20K pH meter (Mettler Toledo) (Rayment and Higginson 1992). For the measurement of organic matter, the dry matter content of sediment was determined after oven-dried at 105 °C for 24 h, then grinded using a mortar and pestle and sieved using a 0.25 mm mesh for the following measurements: sediment ash-free-dry-mass (AFDM) was obtained as the subsequent loss of weight after 4 h at 550 °C in a BF51800 muffle furnace (Thermal) (Hesse 1972). Total organic carbon (TOC), total nitrogen (TN) and total phosphorus (TP) contents were analyzed by the Walkley–Black wet oxidation procedure, the microkjeldahl method and the phosphomolybdic acid blue color method, respectively (Liu et al. 1996). The concentrations of heavy metals including copper (Cu), zinc (Zn), lead (Pb) and cadmium (Cd) were quantified using atomic absorption spectrophotometry after microwave digestion of samples. Briefly, 0.5 g of sieved and dried sediment was added into 9 ml concentrated nitric acid plus 3 ml concentrated hydrochloric acid at 175 °C for 10 min (US EPA 2007). After cooling down, the extracts were centrifuged at 3000 rpm for 5 min; supernatant was analyzed using an AA800 atomic absorption spectrophotometer (PerkinElmer).

### DNA extraction and quantitative PCR

Microbial genomic DNA was extracted from 0.5 g sediment (wet-weight) using a Power Soil DNA extraction kit (MoBio) following the manufacturer’s instructions. The obtained DNA was used as templates for quantitative polymerase chain reaction (qPCR) to determine the copy numbers of bacterial 16S rRNA genes. qPCR was performed with the primer set Eub338F/Eub518R (Fierer et al. 2005). Standard curves ranging from 10^5^ to 10^9^ gene copies per μl were obtained by a tenfold serial dilution of linearized plasmids (Takara) containing cloned 16SrRNA genes that were amplified from *Escherichia coli* DNA. R^2^ value for the standard curves was 0.98, the slope was −3.15, which corresponded to an estimated amplification efficiency of 104 %. All DNA samples were processed along with negative controls and standards.

### Pyrosequencing of bacterial 16S rRNA genes

The V4–V6 region of the 16S rRNA gene was PCR amplified from extracted DNA using universal primers 530F and 1100R (Turner et al. 1999). Both primers contained sequencing adaptor regions; the forward primers also contained sample barcodes (Lu et al. 2015). The PCR program included an initial denaturation at 95 °C for 3 min, followed by 30 cycles of denaturation at 95 °C for 30 s, annealing at 58 °C for 1 min and extension at 72 °C for 1 min and a final extension at 72 °C for 5 min. PCR amplicons were examined by gel electrophoresis (1 % agarose). Verified amplicons were excised from the gels and purified first with a QIAquick gel extraction kit (Qiagen) and then with an Agencourt AMPure XP system (Beckman Coulter). Purified PCR amplicons were quantified using a Quant-iT Picogreen dsDNA Assay kit (Life Technologies). Equimolar amounts of amplicons of different samples were pooled and pyrosequenced in one run using a GS 454 junior sequencing system with unidirectional Lib-L chemistry (Roche 454 Life Sciences) (Lu et al. 2015).

Obtained raw sequence reads were processed using the Pipeline Initial Process of the Ribosomal Database Project (RDP) to sort and rename sequences based on sample tags before trimming off the tags and primers from sequences (Cole et al. 2009). Trimmed sequences were processed using the Mothur software package for quality control and sequence annotations (Schloss et al. 2009). Briefly, sequences that were shorter than 100 bases or contained ambiguous base calls were excluded for further analysis. Subsequently, chimeric sequences were removed. After quality control steps, the remaining sequence reads were clustered into operational taxonomic units (OTUs) at 3 % divergence implemented in Mothur. The longest sequence within each OTU group was assigned as the representative sequence and blasted against the SILVA SSU database for taxonomic annotation (Pruesse et al. 2007). Since bacterial communities were being emphasized in this work, sequences annotated as chloroplasts and archaea were ignored in further analyses.

Pyrotag sequences were deposited in the NCBI Sequence Read Archive (SRA) under the project accession number SRP033375.

### Statistical analysis

Physical and chemical variations among sediment samples were analyzed using principal components analysis (PCA) by Canoco 5.0 (Biometrics). Differences of sediment environmental variables and copy numbers of bacterial 16S rRNA genes among sampling sites were assessed using one-way ANOVA by SPSS 19.0 software package. The level of statistical significance was *p* < 0.05.

Based on taxonomic annotation, sequences were grouped at the order level to construct rarefaction curves and calculate diversity indices (Mou et al. 2013), including Chao1 and Shannon (*H′*) using the Mothur program (Heck et al. 1975). The ∫-Libshuff command in Mothur was used to compare BCCs between sequence libraries. To reveal the bacterial distribution patterns among sampling sites, a heatmap was generated at the order level by PC-ORD5 (MjM Software), based on the same matrix. To investigate relationships between sediment BCCs and environmental variables, distance based redundancy analysis (dbRDA) with Monte Carlo tests was carried out using the Canoco program for Windows 5.0. Furthermore, Pearson coefficient correlations between the major taxa including copy numbers of bacterial 16S rRNA genes, Shannon and Chao1 indices and sediment environmental variables were calculated using SPSS 19.0.

## Results

### Sediment characteristics

**Fig. 2:**
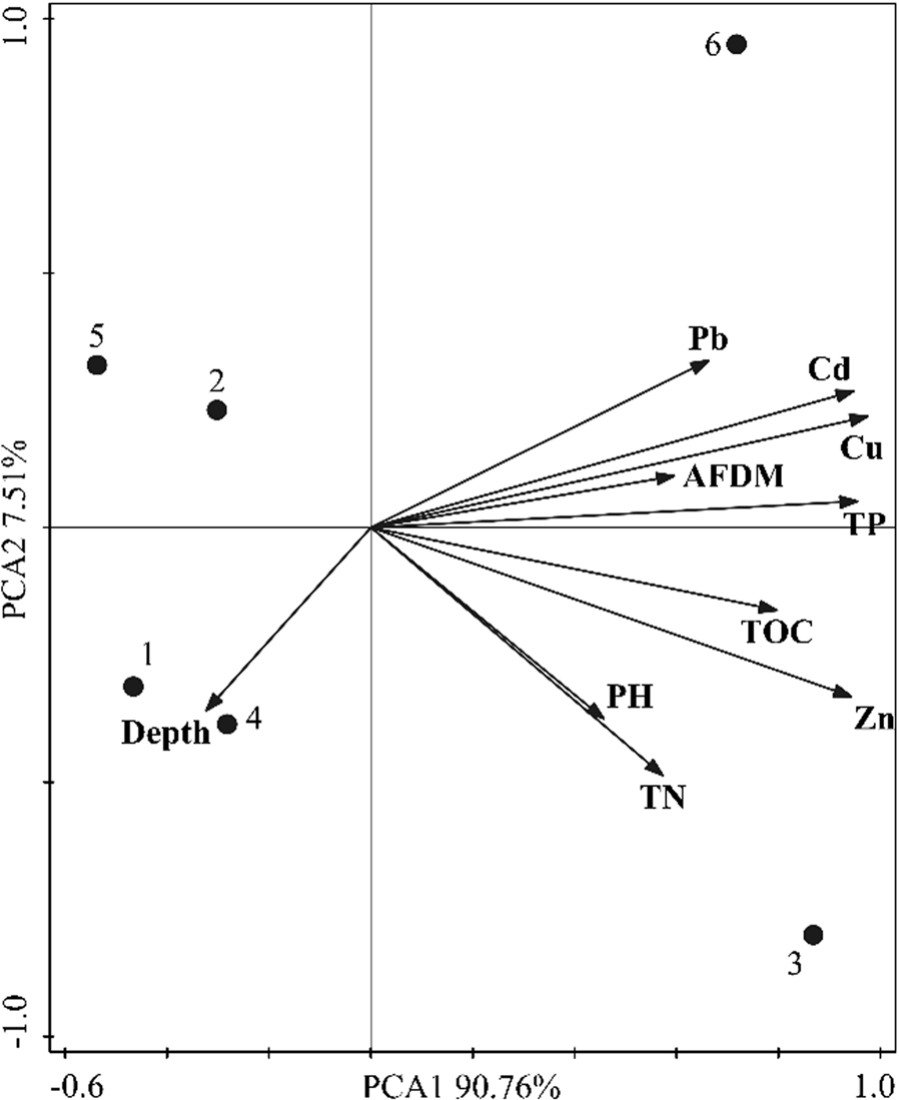
PCA ordination of sediment characteristics

Among the ten tested parameters of Poyang lake sediments, six of which have no difference among samples, only AFDM % (ash free dried mass), TP (total phosphorus), the concentrations of Cu and Cd differentiated (ANOVA, *p* < 0.05) (Additional file 1: Table S1). Principal Components Analysis (PCA) of measured physical chemical variables grouped sites 1, 2, 4 and 5 away from sites 3 and 6. Generally, sites 3 and 6 had greater concentrations of AFDM, TP, Cu and Cd, but shallower water depths than sites 1, 2, 4 and 5 (Fig. 2; Additional file 1 : Table S1).

### 16S rRNA gene abundance

**Fig. 3:**
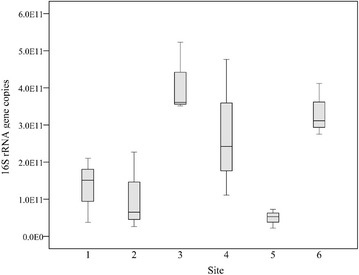
Histogram comparing the copy numbers of bacterial 16S rRNA gene in sediments of Poyang Lake

Quantitative PCR results showed that bacterial 16S rRNA gene abundance varied significantly among six sites (ANOVA, *p* = 0.008). The copy number varied between 4.96 × 10^10^ and 4.12 × 10^11^ copies per gram of dry sediment (Fig. 3), with the higher values found for sites 3 and 6, and the lower values for sites 1, 2, 4 and 5.

### Pyrosequencing statistics and alpha-diversity

The average length of 16S rRNA gene pyrotag sequences (without the primers and adaptors) was 504 bp. A total of 19,892 bacterial 16S rRNA gene sequences were obtained and 18,242 remained after quality filtering and chimera removing. Sequences were grouped into 4394 OTUs (3 % divergence), with 276–1876 OTUs per sample (Table 1). Recovered OTU taxa were affiliated with 25 phyla, 68 orders and 196 species. Rarefaction analysis of bacterial communities was performed at the order level and all but the site 4 library were approaching plateau (Additional file 2: Fig. S1). Order-level Shannon index (*H′*) values were similar for all samples and ranged from 2.96 to 3.21 (Table 1, *p* > 0.05).

**Table 1:** Library coverage estimations and sequence diversity of 16S rRNA genes pyrosequencing

Sampling site	Reads	OTUs	Phylum	Order	Species	*H′*	Chao1
1	3796	1680	22	54	105	3.21	57.00
2	5547	1326	22	54	81	3.02	55.67
3	6422	1876	24	57	115	3.18	59.63
4	427	276	16	40	40	3.02	45.00
5	1034	556	18	42	44	2.96	51.00
6	1016	572	20	44	41	3.07	59.60
Total	18,242	4394	25	68	196		

*H′*, Shannon index

### Bacterial community structure

**Fig. 4:**
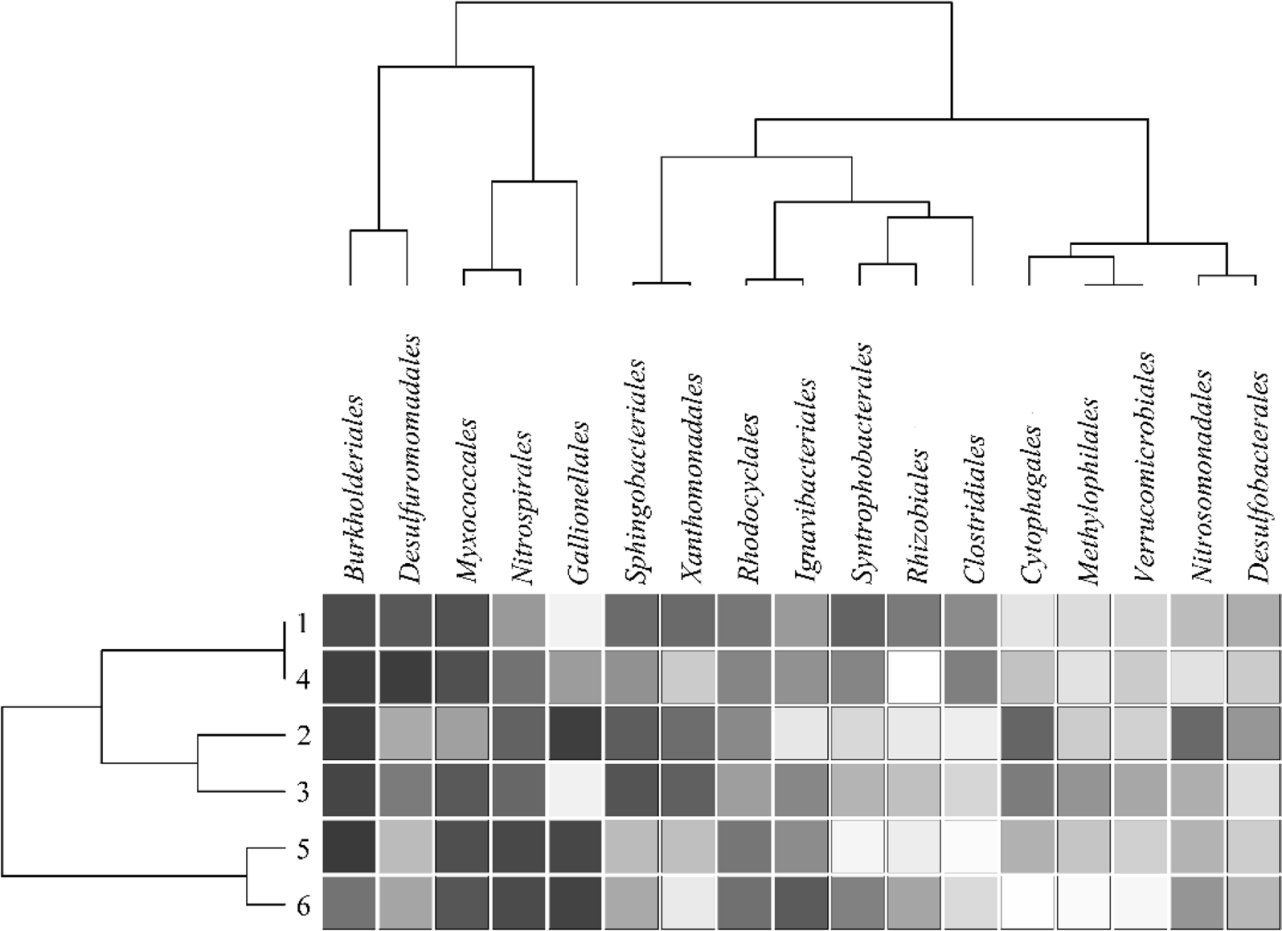
Heatmap analysis of bacterial community composition at order level

Over 83 % of the annotated sequences were affiliated with 17 bacterial orders of 6 phyla. Each of these 17 orders accounted for 2 % or more of the total sequences (Fig. 4) and was designated as major taxa. Except for *Rhizobiales*, all orders were found in each sequence library. *Burkholderiales* was the most abundant taxa and accounted for 12.61 % of sequences on average; it is followed by *Myxococcales* (7.81 %), *Sphingobacteriales* (7.18 %), *Gallionellales* (6.72 %), *Nitrospirales* (6.31 %), *Xanthomonadales* (5.60 %) and *Desulfuromonadales* (5.40 %). *Burkholderiales* represented the most abundant taxa in sediments of all sites except site 6, where *Gallionellales* was the most abundant. *Desulfuromonadales* in the main basin sites (sites 1 and 4; 15.79 and 9.23 %, respectively) were more abundant than those from estuaries (sites 2, 3, 5 and 6, ranged 2.80–4.89 %). *Nitrospirales* occurred mainly in the sites 5 (11.49 %) and 6 (11.09 %) (Fig. 4). Also, heatmap analysis based on relative abundance of major orders grouped samples into three clades: sites 1–4, sites 2–3, and sites 5–6 (Fig. 4). Furthermore, Libshuff analysis based on sequences revealed significant dissimilarities among sequence libraries of the six sites (*p* < 0.0085, with Bonferroni correction) (Additional file 3: Table S2).

Species level annotation was obtained for 3940 sequences (23.5 % of total sequences). Out of the 196 recovered species, *Sideroxydans lithotrophicus* (10.36 %), *Albidiferax ferrireducens* (8.86 %), *Gallionella capsiferriformans* (7.28 %), *Methylobacillus flagellatus* (5.13 %) and *Nitrosospira multiformis* (3.58 %) of *Beta*-*proteobacteria,* and *Geobacter bemidjiensis* (7.89 %), *Anaeromyxobacter dehalogenans* (7.44 %) and *Geobacter lovleyi* (3.88 %) of *Delta*-*proteobacteria* were generally found in all sites (Table 2).

**Table 2:** Main bacterial community compositions in the sediments of Poyang Lake at species level

Sample site	1 (%)	2 (%)	3 (%)	4 (%)	5 (%)	6 (%)
*Sideroxydans lithotrophicus*	3.04	14.68	2.97	8.21	26.67	35.67
*Albidiferax ferrireducens*	1.45	16.12	4.77	5.97	15.19	2.34
*Geobacter bemidjiensis*	11.88	5.87	9.07	13.43	3.70	1.75
*Anaeromyxobacter dehalogenans*	11.74	1.00	10.16	14.18	8.89	14.62
*Gallionella capsiferriformans*	0.00	19.77	0.23	0	1.85	1.75
*Methylotenera mobilis*	1.01	4.23	10.09	1.49	1.85	0
*Geobacter lovleyi*	8.70	1.07	4.22	7.46	2.22	4.68
*Nitrosospira multiformis*	0.00	9.24	0.94	0	0.00	0
*Aquabacterium*	4.49	1.79	4.61	2.24	4.07	0
*Thiobacillus denitrificans*	0.72	0.21	8.60	0.75	0.37	4.09
*Arenimonas oryziterrae*	0.72	4.58	3.83	0.75	0	0
*Geothrix fermentans*	2.32	1.43	1.80	0	4.81	0.58
*Methylobacillus flagellatus*	4.20	0.72	1.33	2.24	4.07	1.17
*Desulfuromonas acetoxidans*	5.94	0.29	0.70	8.96	0.74	0.58
*Sulfuritalea hydrogenivorans*	2.75	0.93	2.35	1.49	0	0.58
*Opitutus terrae*	1.45	2.08	0.86	1.49	2.22	0
*Acidovorax avenae*	0.29	2.01	0.39	0.75	6.67	0

### Influential factors on bacterial communities

Pearson correlation analysis revealed that bacterial 16S rRNA gene abundance was significantly correlated with several sediment property variables, including pH (*r* = 0.54, *p* = 0.02), TP (*r* = 0.70, *p* = 0.001), Cu (*r* = 0.69, *p* = 0.002), Zn (*r* = 0.50, *p* = 0.035) and Cd (*r* = 0.65, *p* = 0.004) (Table 3). Bacterial diversity (Shannon index) or richness (Chao1) was not significantly correlated with any of measured sediment variables (Table 3).

**Table 3:** Correlations between major taxa and environmental variables based on Pearson’s product momentum correlation coefficient

Variables	PH	SM	AFDM	TOC	TN	TP	Depth	Cu	Zn	Pb	Cd
qPCR	*0.537*	*0.702*	*0.615*	*0.761*	*0.639*	*0.698*	–	*0.685*	*0.500*	0.285	*0.649*
*H′*	0.179	0.089	−0.306	0.466	0.603	0.560	0.435	0.284	0.517	−0.339	0.303
Chao1	−0.039	0.500	−0.209	0.269	0.249	0.766	0.195	0.655	0.536	0.329	0.658

Values in italics are different from 0 with a significance level alpha = 0.05

*H′*, Shannon index

**Fig. 5:**
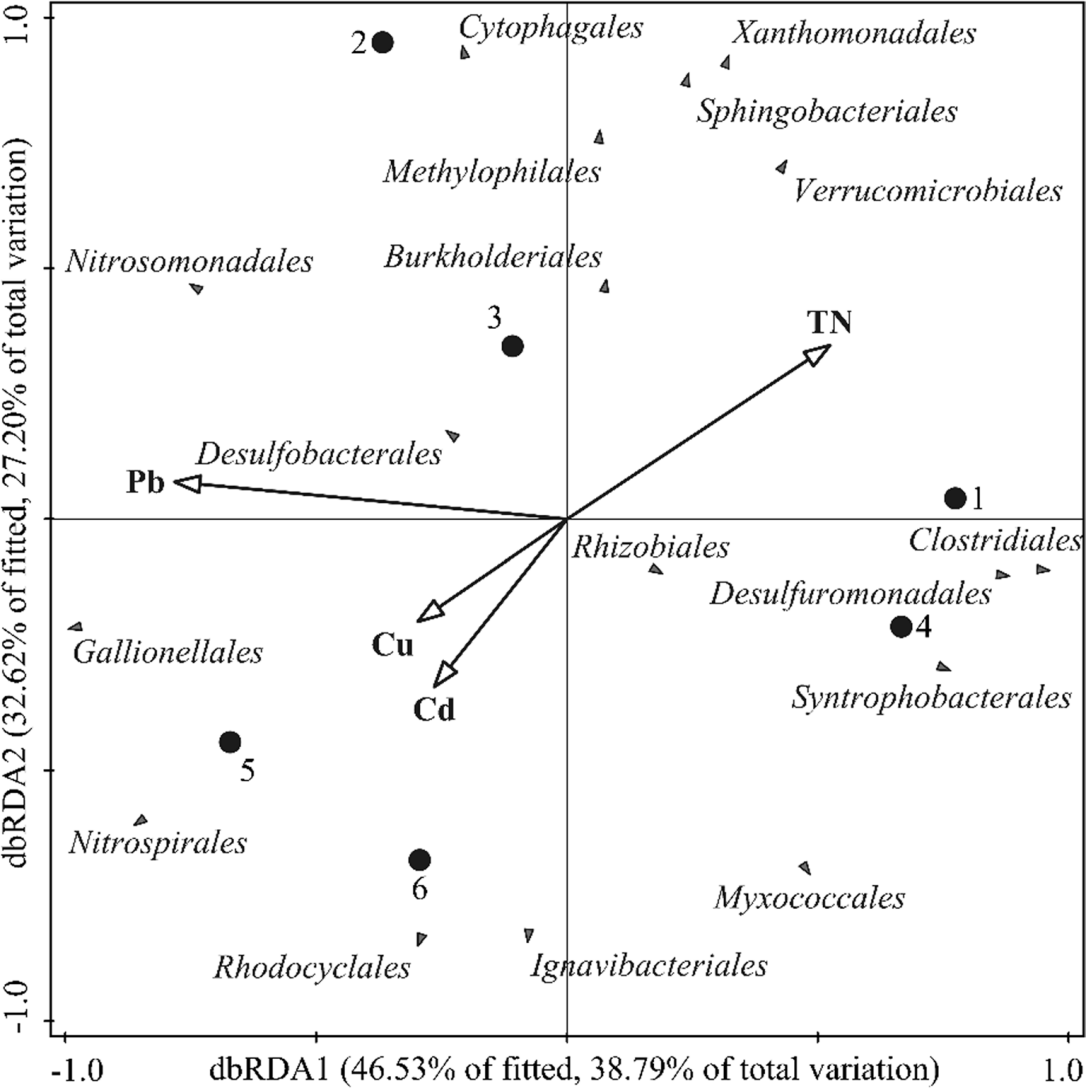
Distance based redundancy analysis (dbRDA) biplots of the sediment bacterial communities associated with environmental variables. *Black* and *solid circles* indicate different samples; *grey triangles* indicate different taxa

To determine the effect of sediment properties on BCC, the property variables were analyzed using dbRDA (Fig. 5), where TN, Pb, Cu and Cd were the most contribution factors as environmental input. Main basin sites (1 and 4) and tributary sites (2, 3, 5 and 6) were mainly separated along the first dbRDA axis, which explained 38.79 % of fitted variation and correlated with TN and Pb contents of the sediment. In addition, the second axis of dbRDA explained 27.20 % of total variation. However, no environmental factors passed the Monte Carlo significance test.

## Discussion

As one of the largest freshwater lakes in China, Poyang Lake provides a number of important ecological services, such as flood storage, regulation of the local climate and habitats for migratory birds. Moreover, it is extremely rich in biodiversity (Wu et al. 2011). However, in recent years, the Poyang Lake has been experiencing the problems of water quality deteriorating and water-level lowering, largely due to the excessive external nutrient loading caused by rapid economic development and agricultural intensification (Wang et al. 2013). The lake ecosystem is facing the degradation trend.

Our study revealed spatial heterogeneity of environmental variables in the sediment of Poyang Lake. For example, contents of TP, Cu and Cd from sites 3 and 6 (Rao River and Xin River estuaries) were significantly higher than other sites (sites 1 and 4 from main basins of lake, sites 2 and 5 from Xiu River and Fu River estuaries) (Fig. 2). This finding is similar to a previous report, in which the concentrations of TP in sediments collected from Rao and Xin River estuaries were higher than those in the center of the Poyang Lake (Wang and Liang 2015). The observed high concentrations of TP, Cu and Cd from Rao and Xin River estuaries are consistent with their locations, which serve as bases for multiple industrial plants such as copper and phosphate mines (Zhang et al. 2014). Our results indicated that Rao River and Xin River were the main input sources of nutrients and metal pollutants of Poyang Lake among the 6 sampling sites and the together appearance of nutrients and metal pollutants were simultaneous.

In accordance with variations in sediment conditions, bacterial abundance and community structure also showed spatial heterogeneity. The 16S rRNA gene copy numbers was 2.31 × 10^11^ copies per gram of dry sediment on average in this study (Fig. 3), similar as those of Taihu Lake, another large and eutrophic lake in China (Ye et al. 2009). Samples of sites 3 and 6 (Rao River and Xin River estuaries) had higher values of 16S rRNA gene copy numbers than the other sites; and this pattern was likely shaped by sediment chemical properties, especially contents of TP, Cu and Cd (Table 3). These findings indicated that high loads of organic compounds and heavy metals may relate to the increases in bacterial abundance. This is in agreement with the results reported in previous studies, which showed positive correlations between sedimentary bacterial abundance and levels of organic matter and nutrients (Steger et al. 2011; Zhang et al. 2015). In addition, bacterial abundance, also recorded using quantitative PCR, found the highest bacterial population within the most heavy metal polluted sediments (Bouskill et al. 2010).

For the spatial distribution of BCCs, heatmap analysis revealed that BCCs of two sites 1 and 4 (Songmenshan and Nanjishan regions) were similar to each other, suggesting similar sediment conditions in these two sites (Fig. 4). This result may be explained by their close positions (both belong to main basin of Poyang Lake) and the sufficient mixing of water current throughout the main basin. However, the BCCs from sites 2 and 3 (Xiu River and Rao River estuaries) were clustered together in Fig. 4, even though their sediment characteristics were significantly different, which were likely affected by riparian inputs or other unknown factors. This result is consistent with the idea that local adaptation maybe is favoring particular lineages in specific regions (Bouzat et al. 2013). Furthermore, our data supported that spatial distribution difference of bacterial communities within a lake is due to shifts in the relative abundance of OTUs rather than variation in presence/absence of some vital species (Staley et al. 2015).

Previous studies reported that nitrogen concentration may have a direct impact on the bacterial composition in both lake water column and sediment samples (Haukka et al. 2006; Zhao et al. 2012). Low levels of Pb contamination in anoxic freshwater sediment (Rush Lake in USA) may impact the community structure of the culturable fraction of the indigenous microbes (Grandlic et al. 2006). In addition, BCCs in Lake Geneva were significantly different between contaminated (High contents of Pb, Cu and Cd) and uncontaminated stations, where sulphate-reducing bacteria and Fe(III)-reducing bacteria (*Geobacter* sp.) were more abundant in the contaminated sediments (Haller et al. 2011). In this work, among pH value, certain compounds (AFDM, TOC, TN, TP, Cu, Zn, Pb and Cr) and water depth, TN and Pb were found to be the most important factors that affected variability of bacterial communities (Fig. 5). However, no significant correlation was observed between sediment physicochemical variables and BCCs. This suggests that BCCs in the sediment of Poyang Lake may be synergistically regulated by multiple factors.

Despite variation among sampling locations, in general, *Burkholderiales* (*Beta*-*proteobacteria*) was the most abundant taxa in the surface sediments of Poyang Lake. This finding is consistent with our previous report in which *Burkholderiales* was found to be dominant in the water column of Poyang Lake (Wu et al. 2012). This taxon is common in freshwater environments (Newton et al. 2011; Staley et al. 2015) and has been found to be able to degrade several aromatic compounds (Pérez-Pantoja et al. 2012). In addition, the observation of abundant *Myxococcales*, *Desulfuromonadales* (*Delta*-*proteobacteria*)*, Sphingobacteriales* (*Bacteroidetes*)*, Gallionellales* (*Beta*-*proteobacteria*) and *Nitrospirales* (*Nitrospirae*) in the sediment of Poyang Lake may reflect the metabolic versatility of these groups. *Gallionellales* was proved to participate in the iron cycle of various waterbody environments (Emerson et al. 2010; Krepski et al. 2012). Therefore, the existence of many iron-oxidizing bacteria affiliated to *Gallionellales* (*Sideroxydans lithotrophicus* and *Gallionella capsiferriformans*, Table 2) demonstrated that the active geological cycle of iron may occur in sediment of Poyang Lake (Blothe and Roden 2009). Nevertheless, the iron content remains to be determined before we are able to draw this conclusion. The prevalence of *Nitrosospira multiformis* in this study revealed active ammonia-oxidizing process, while the presence of *Nitrospirales* members have been known as nitrite-oxidizing bacteria, therefore, nitrification is intensively involved in the nitrogen cycle of lake sediment (Feng et al. 2013; Shen et al. 2013). *Methylobacillus flagellates* occupied almost all samples in this study, and suggested the methyl utilization and carbon cycling in the sediments (Chistoserdova et al. 2007). The predominant *Geobacter bemidjiensis* and *Geobacter lovleyi* represented acetate or tetrachloroethene (PCE) utilization, as well as the reduction of heavy metals (Wagner et al. 2012; Merkley et al. 2015).

## Conclusion

In this study, bacterial abundance and community composition displayed large spatial variations in the sediment of Poyang Lake. Bacterial community abundance was mainly affected by estuaries inputs of both nutrients and pollutants, while bacterial community composition might be affected by the process of biogeochemical transformation. The gradient of nutrients and pollutants, geographically distributed from estuaries inlet to the main basin of Poyang Lake, was formed by estuaries inputs, and afterwards the microbial transformation process followed. Sediment bacterial communities in Poyang Lake were mainly composed of taxa that are typical to freshwater sediment, including *Burkholderiales, Myxococcales, Sphingobacteriales, Gallionellales*, *Nitrospirales*, *Xanthomonadales* and *Desulfuromonadales*.

## Additional files

10.1186/s40064-016-2026-7 Physicochemical variables of sediments in Poyang Lake.
10.1186/s40064-016-2026-7 Rarefaction curves of bacterial pyrosequencing.
10.1186/s40064-016-2026-7 Libshuff comparison between the sequences libraries of samples from six sites.
