# Supplementary material for: Identification of bacterial communities in sediments of Poyang Lake, the largest freshwater lake in China
Source: Springerplus. 2016 Apr 1;5:401. doi: 10.1186/s40064-016-2026-7 (PMC4816951; doi:10.1186/s40064-016-2026-7)
Supplement: Supplementary file 1 — 10.1186/s40064-016-2026-7 Physicochemical variables of sediments in Poyang Lake. [file 40064_2016_2026_MOESM1_ESM.docx]

Table S1 physicochemical variables of sediments in Poyang Lake

| Sampling site | pH | AFDM% | TOC(g/kg) | TN(g/kg) | TP(g/kg) | Depth (m) | Cu(mg/kg) | Zn(mg/kg) | Pb(mg/kg) | Cd (mg/kg) |
| --- | --- | --- | --- | --- | --- | --- | --- | --- | --- | --- |
| 1 | 6.18±0.26a | 3.39±0.43b | 9.30±008a | 0.86±0.01a | 0.62±0.008b | 5.5 | 23.56±0.68b | 155.85±3.62a | 14.63±5.15a | 0.18±0.03c |
| 2 | 6.44±0.18a | 4.80±0.77ab | 7.94±0.05a | 0.86±0.01a | 0.53±0.0002b | 1.5 | 47.66±0.38b | 156.54±2.34a | 55.13±19.76a | 0.12±0.01c |
| 3 | 6.49±0.09a | 5.98±0.16a | 11.90±0.05a | 1.09±0.006a | 0.99±0.0008a | 3.5 | 155.00±0.97a | 297.64±102.10a | 60.60±38.23a | 2.70±0.52b |
| 4 | 6.54±0.33a | 6.74±0.57a | 11.22±0.35a | 0.99±0.05a | 0.49±0.009b | 1 | 37.53±0.44b | 177.13±13.12a | 36.43±30.12a | 0.17±0.02c |
| 5 | 5.99±0.10a | 4.82±0.54ab | 6.62±0.12a | 0.39±0.009a | 0.40±0.009b | 4.5 | 24.45±1.12b | 131.47±40.24a | 47.59±14.74a | 0.11±0.01c |
| 6 | 6.33±0.10a | 6.54±0.55a | 12.28±0.07a | 0.88±0.006a | 1.02±0.003a | 1 | 185.50±21.63a | 225.36±33.67a | 60.54±11.10a | 3.78±0.30a |

Note: AFDM, ash free dried mass; TOC, total organic carbon; TN, total nitrogen; TP, total phosphorus

a, b, c indicate that means were significant different among sampling sites by post hoc-test (*p*<0.05)
