# Supplementary material for: Identification of bacterial communities in sediments of Poyang Lake, the largest freshwater lake in China
Source: Springerplus. 2016 Apr 1;5:401. doi: 10.1186/s40064-016-2026-7 (PMC4816951; doi:10.1186/s40064-016-2026-7)
Supplement: Supplementary file 2 — 10.1186/s40064-016-2026-7 Rarefaction curves of bacterial pyrosequencing. [file 40064_2016_2026_MOESM2_ESM.docx]

**Fig. S1** Rarefaction curves of bacterial pyrosequencing

Note: 1-6 represent samples from different sites
