# Supplementary material for: Identification of bacterial communities in sediments of Poyang Lake, the largest freshwater lake in China
Source: Springerplus. 2016 Apr 1;5:401. doi: 10.1186/s40064-016-2026-7 (PMC4816951; doi:10.1186/s40064-016-2026-7)
Supplement: Supplementary file 3 — 10.1186/s40064-016-2026-7 Libshuff comparison between the sequences libraries of samples from six sites. [file 40064_2016_2026_MOESM3_ESM.docx]

Table S2 Libshuff comparison between the sequences libraries of samples from six sites.

| Comparison | dC_XY_Score | Significance (*p*) |
| --- | --- | --- |
| 1-2 | 0.00493561 | <0.0001 |
| 2-1 | 0.01403633 | <0.0001 |
| 1-3 | 0.00071966 | <0.0001 |
| 3-1 | 0.004233 | <0.0001 |
| 1-4 | 0.01181458 | <0.0001 |
| 4-1 | 0.00154953 | 1 |
| 1-5 | 0.00789249 | <0.0001 |
| 5-1 | 0.00042239 | 1 |
| 1-6 | 0.01040038 | <0.0001 |
| 6-1 | 0.0006476 | 1 |
| 2-3 | 0.00885529 | <0.0001 |
| 3-2 | 0.00865158 | <0.0001 |
| 2-4 | 0.03005456 | <0.0001 |
| 4-2 | 0.00025209 | 1 |
| 2-5 | 0.01561119 | <0.0001 |
| 5-2 | 0.00061157 | 1 |
| 2-6 | 0.02468377 | <0.0001 |
| 6-2 | 0.00158204 | 1 |
| 3-4 | 0.02023094 | <0.0001 |
| 4-3 | 0.00163879 | 1 |
| 3-5 | 0.01245685 | <0.0001 |
| 5-3 | 0.00032377 | 1 |
| 3-6 | 0.01643219 | <0.0001 |
| 6-3 | 0.00020758 | 1 |
| 4-5 | 0.00044357 | 0.9996 |
| 5-4 | 0.00665833 | <0.0001 |
| 4-6 | 0.00151733 | 0.6584 |
| 6-4 | 0.00935104 | <0.0001 |
| 5-6 | 0.00386535 | <0.0001 |
| 6-5 | 0.00353855 | <0.0001 |

Note: 1-6 represent samples from different sites
